# Activation of Endogenous Retrovirus, Brain Infections and Environmental Insults in Neurodegeneration and Alzheimer’s Disease

**DOI:** 10.3390/ijms22147263

**Published:** 2021-07-06

**Authors:** Federico Licastro, Elisa Porcellini

**Affiliations:** Department of Experimental, Diagnostic and Specialty Medicine (DIMES), University of Bologna, 40126 Bologna, Italy; Elisa.porcellini3@unibo.it

**Keywords:** Alzheimer’s disease, inflammation, human endogenous retrovirus activation

## Abstract

Chronic neurodegenerative diseases are complex, and their pathogenesis is uncertain. Alzheimer’s disease (AD) is a neurodegenerative brain alteration that is responsible for most dementia cases in the elderly. AD etiology is still uncertain; however, chronic neuroinflammation is a constant component of brain pathology. Infections have been associated with several neurological diseases and viruses of the Herpes family appear to be a probable cause of AD neurodegenerative alterations. Several different factors may contribute to the AD clinical progression. Exogeneous viruses or other microbes and environmental pollutants may directly induce neurodegeneration by activating brain inflammation. In this paper, we suggest that exogeneous brain insults may also activate retrotransposons and silent human endogenous retroviruses (HERVs). The initial inflammation of small brain areas induced by virus infections or other brain insults may activate HERV dis-regulation that contributes to neurodegenerative mechanisms. Chronic HERV activation in turn may cause progressive neurodegeneration that thereafter merges in cognitive impairment and dementia in genetically susceptible people. Specific treatment for exogenous end endogenous pathogens and decreasing pollutant exposure may show beneficial effect in early intervention protocol to prevent the progression of cognitive deterioration in the elderly.

## 1. Retrotransposons and Human Endogenous Retrovirus in Neurodegenerative Diseases

Retrotransposons constitute approximately 40% of the human genome and are major drivers of genomic evolution [1,2,3]. Three main classes of retrotransposons have been described in humans: long interspersed nuclear elements (LINEs; ∼17%), short interspersed nuclear elements (SINEs; ∼10%), and long terminal repeat retrotransposons (LTRs; ∼8–10%) [1,2].

It has been reported that approximately 0.1% of spontaneous mutations are caused by retrotransposon insertion and ∼95% these mutations are induced by SINE or LINE [4,5].

**Figure 1:**
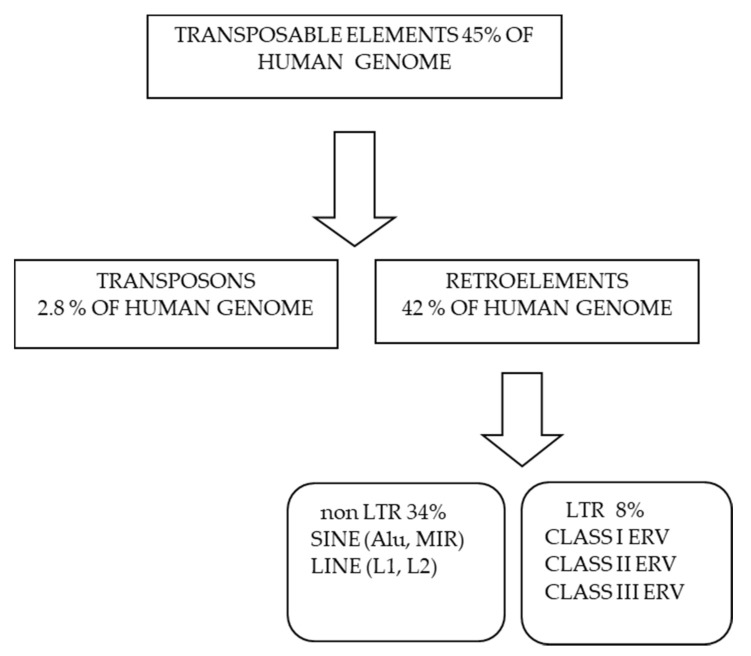
Classification of transposable elements and their relative abundance in human genome.

A classification of transposable elements in human genome is reported in Figure 1. Up to 8% of the human genome has a retroviral origin. These ancient retroviruses, now called human endogenous retroviruses (HERVs), invaded the germ line of our primate ancestor millions of years ago and were subsequently integrated into their genome [6]. Retrotransposon activity is responsible for the amplification of these integrated retroviral elements (ERV) [7], and most retroviral genes contain deletions or nonsense mutations being therefore silent. However, some ERV maintained partial functionality and developed into enhancers of the immune genes [8].

For instance, syncytin is encoded by HERV-WE1, a full-length provirus at locus 7q21.2 on chromosome 7 and is involved in placental development [9]. HERV elements are normally expressed at low levels, however, hypoxia [10], drugs [11], infections by exogeneous viruses [12], and some mutations [13] can increase their expression. Another inductor of ERV is inflammation, which plays a major role in HERV activation [14,15,16].

The mammalian genome has evolved molecular mechanisms [17] to control the autonomous activation of retrotransposons; some of these overlap with antiviral immune defenses.

For instance, DNA methylation [18,19,20,21], nucleic acid sensing Toll-like receptors (TLRs) [22], cytosolic and lysosomal DNases [23,24], and immunoglobulins [25,26] are known control mechanisms. However, disruption or impairment of these mechanisms may induce de-repression of retrotransposons which are associated to several human and animal pathologies, such as autoimmune diseases and cancer [18,19,22,23,24,25,26]. For instance, the activation and even transmissibility of ERVs has been reported in high leukemic mouse strains [27,28,29].

Few mutations associated with human retrotransposon activation have been implicated in familial neurodegenerative syndromes and some autoimmune disorders [23,30,31,32].

An abnormal activity of HERV has also been described in human brain diseases. For instance, levels of HERV-K have been found elevated in patients with amyotrophic lateral sclerosis [31,32,33,34]. On the other hand, increased HERV-W activity has been described in human multiple sclerosis [35,36]. Regarding the topic of this paper, it is of interest that increased LTR and non-LTR retrotransposon have been associated with Alzheimer disease [37]. Finally, a recent paper showed that ERV activation was associated with hippocampus-related learning deficits in mice and concluded that these cognitive effects were mediated by the cytosolic RNA sensor MAVS [38].

The above data are suggestive that ERV activation might represent a mechanistic and pathogenetic link of inflammation and neurodegeneration with some human disease and brain aging and pathology. However, our knowledge regarding the inducers of retrotransposon and ERV activation is still limited.

In this paper, we illustrate and discuss a new notion suggesting that limited and chronic insults of central nervous system (CNS) such as infections by microorganisms or insults by pollutants may activate previously silent endogenous retrovirus which in turn may induce chronic inflammation, amyloid production, and neuro degenerative pathology associated with AD.

## 2. Alzheimer Disease, Chronic Inflammation, the Amyloid-β, and Virus Infection

An increasing burden of neurological disorders has been reported in the USA, with stroke, Alzheimer disease (AD), and other dementias being prevalent neurological diseases [39].

AD is a chronic neurodegenerative disease and the prevalent form of dementia in the elderly. Declining immunity during ageing is often associated with peripheral chronic inflammation [40] and chronic neuroinflammation is a constant component of AD brain pathology [41].

Several alleles in immunoregulatory genes have been shown to modify the AD risk and most of these gene are also involved in immune defenses against viruses and other microbes [42].

Pathogens, such as viruses of the Herpes family, through frequent cycles of reactivation and latency, constantly trigger the immune system; however, immune responses cannot completely eradicate these microbes. Therefore, we suggested that these persistent neurotropic pathogens might play a role in microglia activation in the brain of genetically susceptible elderly and promote neurodegenerative processes [42].

The amyloid-β (Aβ) peptide is associated with AD neurodegenerative processes and show antimicrobial activity against eight common and clinically relevant microorganisms [43]. Moreover, Aβ peptide shares several chemical and biological characteristics of antimicrobial peptides (AMPs) which are components of the innate immune system [43].

The Aβ peptide has also been shown to play a protective function against in vitro infection by Herpes Simplex virus-1 (HSV-1) [44]. This observation reinforces the notion that persistent and latent Herpes virus infections of human brain may lead to Aβ peptide overproduction and amyloid plaque formation [44]. Mutations of human amyloid protein might interfere with normal brain or systemic immune defenses, however, at the present it is not known whether APP mutations might affect control of transposons and HERV activity.

Recent findings have confirmed a causative role of viruses of the Herpes family in AD [45] and a relevant relationship of Human Herpes virus 6 (HHV-6) and 7 (HHV-7) with the disease. However, discordant data regarding a pathogenetic link of HHV-6 and HHV-7 with AD are also on record [46,47]. Therefore, the association of these viruses with the disease deserves further investigation.

Our recent data showed that antimicrobial defense mechanisms of innate immunity appeared to be impaired in AD brains and we suggested that these immune alterations might contribute to neurodegeneration [48].

However, findings regarding the presence of viruses or other pathogens in human normal and AD brains are conflicting and a pathogenetic role of infectious agents in AD pathogenesis remains controversial.

## 3. Tau Protein and Jumping Genes

Tau intra neuronal deposition is a neurodegenerative hallmark of AD and tau pathology is also found in a heterogeneous group of neurodegenerative syndromes, the tauopathies, causing cognitive and/or motor impairment.

Chromosomal instability is frequently found in ageing and neurodegenerative diseases and AD brain pathology is accompanied by genomic instability in affected neurons [49].

In a drosophila model, tau induces global nuclear chromatin relaxation [50], abnormal transcriptional activation of heterochromatic genes, and DNA double-strand breaks [51].

Recently, differential expression for several retrotransposons in association with neurofibrillary tangle burden was observed in serial RNA sequencing from human AD brains [37]. These results highlight evidence for global transposable elements (TE) transcriptional activation among the LINE-1 and ERV clades I. The authors concluded that their results implicate TE activation, along with the presence of genomic instability associated with tau-mediated AD mechanisms.

Moreover, another investigation provided evidence that the tau protein promoted neuronal death in tauopathies by dysregulating TE [52].

Recently, a study showed that the plasma levels of a phosphorylated form of tau (p-tau217) increased over time in preclinical AD, e.g., cognitively unimpaired subjects with brain amyloid deposits, and prodromal stages of AD or MCI patients with brain amyloid deposits. Moreover, the plasma levels of p-tau217 increased with longitudinal worsening of cognitive performances and brain atrophy [53].

These results might reinforce the notion of a pathogenetic role of tau in AD. However, we previously discussed that tau may induce HERV activation and neurodegenerative related mechanisms. Therefore, elevated p-tau217 appears to be a useful marker of cognitive impairment and might also be associated with TE dysfunction in AD. This topic deserves further studies.

## 4. Neurotropic Exogenous Viruses and HERV Dysregulation in Neurodegeneration and AD

Currently, two exogenous retroviruses are known to induce human diseases: human immunodeficiency virus (HIV) and human T lymphotropic virus (HTLV). While HTLV is a “classical” oncovirus, causing T cell leukemia, HIV infection causes acquired immunodeficiency syndrome (AIDS), which is accompanied by several comorbidities, including an increased incidence of some cancers [54]. Viruses of the *Retroviridae* family contain two copies of single-stranded RNA (ssRNA) genome at their core and are encapsulated by host-derived lipid membrane inserted with the surface gp120 and the transmembrane gp41 proteins [55]. In general, retroviruses can perform reverse transcription of their ssRNA genome and integrate it into the host chromosomal DNA [56].

On the other hand, ERVs are considered molecular remnants of ancient exogenous retroviruses which integrated their genomes in vertebrate ancestors during evolution [57].

Here, we suggest that neuro infection agents such as exogenous neurotropic virus, other human pathogens with neuro damage potential, and chemical environmental insults might cause ERV activation by inducing brain temporary immune responses and inflammation. For instance, cyclic endogenous re-infections by virus of the Herpes family and chronic insults by environmental pollutants might be particularly relevant in inducing retrotransposon mutations and ERV induction. Virus–virus interactions have been reported in the pathogenesis of several human diseases. Examples are coinfection of hepatitis C virus (HCV) with HIV [58] or other hepatitis viruses [59]. Other examples are coinfections of HIV and HHV-6 [60] or HHV-7 [61].

Results regarding HHV-6A and EBV interaction with HERVs, as a pathogenic mechanism in multiple sclerosis (MS), have also been reported [62]. Those data showed that HHV-6A and EBV induced HERV-K18-encoded superantigen and host T-cell responses to such super antigen could secondarily lead to local autoimmune phenomena.

Finally, data are on record showing that the HIV Rev protein can functionally interact with many repetitive elements (RcREs) present in the human genome, depending on the RcRE sequence, as well as the Rev sequence. This interaction may lead to the export of some of the HERV-K pro-viral mRNAs, changing the expression of non-viral genes [63].

HERVs’ expression depends on several factors, and is epigenetically regulated by stimuli such as inflammation, viral and microbial infections. Increased expression of HERVs occurs in physiological and pathological conditions and several diseases have been attributed to one or more HERVs, particularly neurological diseases.

We suggest that microbes’ infections of human CNS not only may directly induce inflammation and neurodegeneration, but they may also activate brain HERV which in turn by acting as continuous abnormal stimulation of inflammatory responses may contribute to neurodegenerative hallmarks associated with AD.

## 5. HERV Dysregulation AD and Neurological Diseases

Our hypothesis is supported by data regarding abnormal retrotransposon activation in AD which are indeed on record. For instance, a quantitative bisulfite-PCR pyrosequencing method was used to evaluate the methylation of Alu, LINE-1 and SAT-α sequences in 43 AD patients and 38 healthy donors and LINE-1 methylation resulted increased in AD patients [64].

Moreover, Guo et coworkers [37] leveraging RNA sequencing from 636 human brains, found differential expression for several retrotransposons which resulted associated with neurofibrillary tangle burden. The authors also showed evidence for global TE transcriptional activation among the long interspersed nuclear element 1 and endogenous retrovirus clades. Finally, they detected tau-associated, active chromatin signatures at multiple HERV-Fc1 genomic loci. Their results show that TE activation is involved in tau neuropathology and tau-mediated AD mechanisms were associated with genomic instability able to induce brain TE activation.

The above results were confirmed and extended, since TE dysregulation was identified as a key mediator of neuronal death in tauopathies, and a significant increase in HERVs transcripts was found in AD and in progressive supranuclear palsy, suggesting that TE dysregulation might be a hallmark in human tauopathy [52].

Recently, the increased brain expression of ERV has been shown in a drosophila model of human frontotemporal dementia (FTD) which is the second most prevalent form of human pre-senile dementia after AD. It was found than neuronal expression of CHMP2BIntron5 causes an increased activity of the endogenous drosophila RV, called gypsy, in the nervous system. Genetically blocking Drosophila gypsy activation or pharmacologically inhibiting viral reverse transcriptase activity stopped degenerative phenotypes observed in fly and rat neurons [65].

Since FTD appears to overlap genetically and pathologically with amyotrophic lateral sclerosis (ALS), the authors [65] claimed that their observations may also further contribute to previous discoveries of HERV activation in ALS affected patients.

Whether microorganism infections might induce the activation of ERV and whether such an activation might play any role in the pathogenesis of human diseases deserve more investigations. However, results are on record suggesting that immune cells activation by microbes induced increased expression levels of endogenous ERV [66]. The authors, by employing a microarray-based method that allows a broad determination of ERV expression, found extensive patterns of ERV modulation by commensal or pathogenic microbes in both murine and human tissues. They concluded that ERV responsiveness to external stimuli such as microbe infections or dysbiosis should be considered in any association between ERV transcription and human diseases [66].

Infection with virus of the Herpes family can also induce an increased expression of ERV, as showed in a mouse model after infection with EBV. In this animal model lymphocytes from infected animals showed increased transcription of the env gene of HERV-K18. This protein is known to possesses a super antigen activity [67] and may induce autoimmune response. HERV activation may also be induced by other pathogens. In fact, an in vitro infection of peripheral blood leukocytes with coxsackie virus-B induced high levels of the HERV-W env mRNA [68].

It of interest that EBV, even during the latent phase of the infection, appears to be able to induce ERV activation in men. In fact, it has been reported that activation of MSRV-type of ERV was detectable during both human infectious mononucleosis and EBV latency [15].

In this context, EBV glycoprotein 350 (EBVgp350) was found to trigger the expression of HERV-W *env* in blood cells and astrocytes, and these mechanisms might contribute to the onset of MS [15]. Similar activation mechanisms on HERV were demonstrated for Herpesviridae HSV-1 and HHV-6 [69,70,71,72] providing evidence for underlying common virus activities and explaining the well-established epidemiological link between infections by these viruses and the susceptibility for MS.

An important activator of HERV expression is the nuclear factor kappa-light-chain-enhancer of the activated B-cells (NF-kB) signaling pathway. These data are based on an earlier report demonstrating that the pro-inflammatory cytokine tumor necrosis factor (TNF) can stimulate the HERV-W promoter, at that time known as ERVWE1/syncytin, via the induction of NF-kB [14].

A summary of the concomitant presence of exogenous virus and HERV activation in human neurological disease is presented in Table 1.

Therefore, an initial inflammation of small brain areas induced by Herpes virus invasion may induce a dysregulation of HERV activation and expression which in turn may contribute to neurodegenerative mechanisms. Subsequent virus cycles of activation and latency may induce not only limited brain damages and neurodegenerative mechanisms, but also persistent HERV activation which in turn may cause further neurodegeneration. These vicious and damaging cycles thereafter merge in cognitive impairment and dementia in genetically susceptible people.

### 5.1. Amyloid and Tau Protein in Exogenous Virus Infection and HERV Activation

Amyloid deposition and the accumulation of iper-phosphorylated Tau (p-Tau) are two classical hallmarks of AD neuropathology. However, accumulating evidence have shown that these two compounds are also induced by infection with exogenous virus both in vivo and in vitro models. For instance, it was shown that infection of cultured neuronal and glial cells with HSV-1 leads to a dramatic increase in the intracellular levels of A-β peptides 1–40 and 1–42, [73].

The observation was then confirmed in a in mouse model where the HSV-1 repeated cycles of activation, latency, and reactivation were induced by thermal stress. In this animal model, the accumulation of AD hallmarks including amyloid-β protein, tau hyperphosphorylation, and neuroinflammation markers such as astrogliosis, IL-1β, and IL-6 were induced. Moreover, repeated virus reactivations correlated with increasing cognitive deficits which become irreversible after seven cycles of infection [74]. It of interest that the reactivation of HSV-1 in mice also induced impaired adult neurogenesis in the sub-granular zone of hippocampal dentate gyrus. [75]. These data showing that repeated cycle of virus activation was deleterious for hippocampal functioning, implied that virus lesions in the human brain might play a role in AD development.

Different viruses of the herpes family, such as HHV-6 and 7, were also able to seed A-beta deposition in a mutant mouse model of AD (5xFAD) or in a 3D model of human neural cell cultures. The author suggested that amyloid deposition leads to protective virus entrapment [76].

Recent data showed that p-tau was transiently induced in mouse adult hippocampal neuron cultures infected by HSV-1, whereas A-beta was constantly produced during infection and colocalized with HSV-1 latency-associated transcript (LAT). The authors concluded that p-Tau may act as acute phase response factor, while A-beta aggregation may represent a long-term response to infection. [77].

However, discordant results are on record suggesting that amyloid may not be protective against virus infection in the 5xFAD animal model of AD. [78].

A relationship between brain infection and amyloid and Tau protein is not limited to viruses of the Herpes family. HIV, a human exogenous retrovirus, also show the ability to induce amyloid production and deposition in the brain of patients with HIV-associated neurocognitive disorder (HAND) [79]. Recently was shown that HIV duration and not the active brain infection correlated with brain amyloid deposition [80].

The above results support the notion that infection by exogenous viruses may indeed induce brain A-β peptide production, amyloid deposition, and increased p-tau as counteracting antimicrobial mechanisms.

Data regarding the role of amyloid or tau proteins in HERV activation and control are scarce. However, when HERVs are activated, they act as infectious virus and induce cell invasion and inflammatory response. Therefore, HERVs may contribute to neurodegeneration by chronic inflammatory pathway activation in human brain.

Recently, it was reported that amyloid deposition in mouse hippocampus induced increased SINE B2 non-coding RNA processing. [81]. On the other hand, it was shown that tau can promote neurodegeneration by inducing global chromatin relaxation [50]. Chromatin relaxation in turn may induce TE and HERV activation and promote further neurodegenerative processes.

Recent data showed that increased HERV transcripts were detectable in the brains of patients with different tauopathies [52]. These data indirectly support a link of abnormal tau expression and deposition and HERV activation with human brain neurodegeneration.

On the other hand, the inappropriate activation of HERVs might interfere with brain immune response against exogenous virus and other microbes and increase the invasive behavior of these pathogens.

In conclusion, a non-casual relation appears to link amyloid and tau proteins to infection by exogenous virus and abnormal expression of transposons and HERV in brain affected by neurodegenerative processes.

### 5.2. Environmental Pollution and Neurodegeneration

Pathogens are not the only insults able to induce neurodegenerative lesions in the human brain. In fact, pollutants can induce many neuro-pathological alterations and affect brain functioning. A recent report indicates that in a cross-sectional study of 18,178 individuals with cognitive impairment, people living in areas with worse air quality were more likely to have positive amyloid positron emission tomography scan results [82].

Moreover, higher particulate (PM_2.5_) air levels appeared to be associated with brain amyloid-β plaques, one neuropathological hallmark of AD [82]. Therefore, exposure to air pollution is associated with amyloid-β pathology in older adults and increases the risk of cognitive impairment and AD.

It is of interest that acute and chronic exposure to PM has been shown to induce differential methylation of EBV in heavy steel workers [83]. The authors concluded that the difference observed comparing baseline and post-exposure samples may be suggestive of a rapid change in EBV methylation induced by air particles [83].

An interesting longitudinal investigation assessed the relationship of long-term exposure (2001–2004) to air pollutants (PM_2.5_ and NOx) and concomitant presence of cardiovascular disease on the risk of developing dementia in a selected population from Sweden. The study found that air pollution exposure was associated with a higher risk of dementia. Patients suffering heart failure and ischemic heart disease and exposed to air pollutants showed increased risk of dementia, whereas stroke seemed to be an important intermediate condition between the association of air pollution exposure with dementia. [84]. Air pollutants appear to induce multiple tissue damage and increase the incidence of both cardiovascular diseases and dementia.

An epidemiological investigation on a different population resident in Hon Kong also found that long term exposure (1998–2011) to PM_2.5_ increased the incidence of both AD and vascular dementia [85]. All together these data indicate that air pollution affects AD and dementia risk in different racial populations.

An experimental investigation using an AD mouse model (APP/PS1 transgenic mice) exposed for three months to PM_2.5_ showed that brains from these animals had increased levels of amyloid plaque deposition, astrogliosis and inflammation [86]. Therefore, air pollutants can reach the brain and contribute to neurodegenerative alterations associated with AD.

Chemical pollutants are also involved in brain damage. In fact, another study using APP/PS1 transgenic mice exposed to benzo(a)pyrene for two months showed that brains from these animals suffered increased neuronal loss, A-β peptide burden and amyloid deposition [87].

Chronic insults from air and chemical pollutants reaching the brain may also interfere with transposons and ERV activation and control.

Preliminary results are on record suggesting that another environmental chemical pollutant such as bisphenol A (BPA) activates HERV [88]. Moreover, the human placenta appeared to be a target organ of BPA. In fact, at 50 nM, BPA induced ERVW-1, ERVFRD-1 and expression of the corresponding syncytin proteins, ERV3-1, PPARγ, ERα and ERβ. BPA also increased β-hCG secretion and BeWo cells fusion, thus promoting the syncytial-trophoblast phenotype [69].

Results reported above suggest that chronic insults such as air and chemical pollutants induce brain damages, promote AD associated neurodegenerative hallmarks and in some instance may activate HERVs and induce dysregulation of RE which might have pathogenetic consequences on neurodegenerative mechanisms.

Alternatively, we cannot exclude that environmental pollutants may induce neurodegenerative processes by activating latent human neurotropic virus such as those of the Herpes family which in turn are able to induce AD related neuropathology directly or after the activation of HERV.

## 6. SARS-Cov-2 and Cognitive Impairment

Another emerging human virus with brain lesion potential is the pandemic SARS-Cov-2 or COVID-19 virus. This virus infects nasal mucosa and the respiratory tract causing in several instances severe respiratory distress and death in frail patients.

COVID-19 virus along with other neurotrophic virus, such as those of the Herpes family, can enter the brain by olfactory fibers of the nasal mucosa and the olfactory nerve, thereafter reaching by axonal retrograde transportation the human brain. Therefore, neurotropic virus may access the brain by olfactory mucosa and its nerve terminations. On the other hand, air pollutants might use the same way to enter human brains. Here, these insults may directly induce inflammation and neurodegenerative mechanisms and/or indirectly promote neuronal damages by activating HERVs which may induce chronic brain inflammation and neurodegeneration.

COVID-19 is indeed a neurotropic virus and can induce cognitive impairment and brain alterations during the acute clinical phase. It is of note that COVID-19 infection accelerates cognitive deterioration in persons with preexistent cognitive impairment and conversion of patients with mild cognitive impairment to AD [89]. This neuropathological effect of COVID-19 infection was particular evident in the elderly living in institutes for old people [90].

The spike protein of COVID-19 has been shown to bind amyloid peptide and tau protein and increase deposition of these molecules [91]. Moreover, a recent investigation focused on neuropathological brain hallmarks from persons died of COVI-19 reported the presence of focal β-amyloid precursor protein immunoreactivity in white matter. [92]. These observations imply an interference of COVID-19 with neurodegenerative proteins and open the question regarding its potential effect on risk of cognitive decline in long term survivors from COVID-19 brain infection.

Whether this virus might induce a dysregulation of HERV in the brain and long-term alterations of brain metabolism also remain open questions.

### Smell, Virus Infection and Neurodegeneration

Olfaction is an animal ancient sense. Across different species, it modulates the interactions between an organism and the surrounding environment. In different neurological pathologies, like Parkinson’s and Alzheimer’s disease, dysosmia is exacerbated compared to the physiological decrease associated with ageing and occurs before motor and cognitive disabilities [93].

Many studies claim that the alteration of the olfactory system may be used as an early predictor for detecting AD, because several brain olfactory regions are impaired in the asymptomatic prodromal phase of this disease due to the deposition of pathological hallmarks [94].

We have already discussed that acute virus infections, such as COVID-19, as well as air pollutants are able to damage olfactory mucosa and its nerve terminations. Therefore, it not surprising that the decline in smell acuity and sensitivity is also an early sign of acute COVID-19 virus infection and possibly of chronic persistent infection by some viruses of the Herpes family.

It remains to be determined whether smell impairment may correlate with recently reported blood markers of impaired cognition such as p-tau217 and used in tandem to clinical screening of cognitive impairment in pre-clinical phases of dementia.

## 7. Conclusions

As presented here, environmental insults, such as persistent virus infection and pollutants, may reach the brain via anterograde transportation along the olfactory nerve and induce neuron damages which in turn may activate HERV and TE disfunction.

This hypothesis is compatible with our recent data showing impaired mRNA expression of IFN pathway in AD brains, since both exogeneous viruses and HERV induce IFN defensive mechanisms [48].

The activation of HERV may induce neuronal infection and intraneuronal damages amplifying neurodegenerative alterations and activation of persistent neuroinflammation with further neuronal damages.

**Figure 2:**
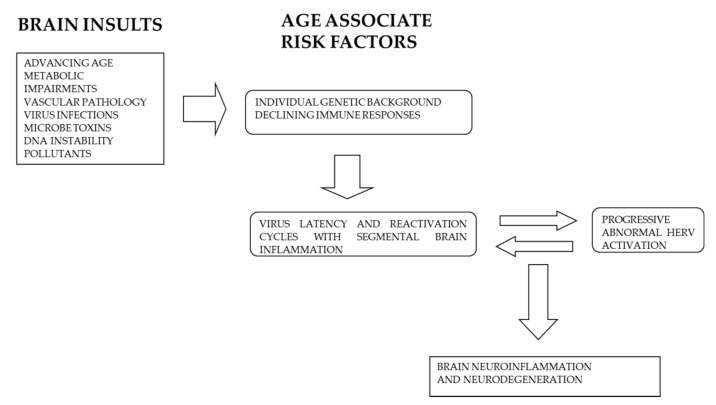
Different age-related risk factors contribute to the interplay between exogenous virus infections and HERV abnormal activation leading to neuro-degenerative processes and AD.

Inappropriate chronic activation of HERV induced by infections or other environmental insults may represent the driving force able to induce continuous neurodegenerative processes that after several years may merge as clinical manifestation of dementia and other neuro-degenerative diseases. Figure 2 summarizes the different pathogenetic steps which may lead or contribute to neurodegenerative alterations associate with AD.

An inappropriate activation of HERV may induce neurodegenerative processes by different mechanisms. First, these ancient retroviruses can induce inflammation and immune responses mediated by Toll-like recognition receptors and type I-IFN production. Therefore, HERV by inducing neuroinflammation contribute to neuronal and synaptic loss. Second, HERV activation or their induced inflammatory response may activate other transposable elements which in turn may induce neuronal loss by increasing chromatin instability.

Therefore, HERV activation might be the missing link between exogenous brain insults and internal damaging mechanisms such as A-β peptide and Tau expression and deposition. Other factors such as age, gender, genetic makeup, smoke, exercise, and diet also contribute to neurodegenerative processes.

The notions presented here have relevant implications for the prevention and treatment of prodromal and early phase AD.

In fact, it is possible to diagnose the presence of Herpes virus infection and reinfection by detecting plasma levels of IgM and IgG virus specific antibodies. In subject with high level of these antibodies anti-viral treatment would be advisable. Following up these persons and monitoring their cognitive performances during and after anti-viral treatment would be also advisable and would provide valuable information.

It is of interest that a randomized clinical double blind clinical trial of AD patients with valacyclovir, an anti-herpetic drug, is in progress [95].

Moreover, infections by exogeneous viruses can induce activation on HERV and we discussed the potential serious consequences of HERV reactivation on cognitive decline and dementia risk.

Several laboratory analyses are available to detect HERV activation and infection and some antiretroviral drugs have been proposed for specific treatment of these new potential infectious agents [96]. Antiretroviral drugs have been indeed used for the treatment of HERV infection in patients with multiple sclerosis [97].

We also suggest the relevance of detecting the possible presence of other pathogen infections in prodromal and early clinical phase AD and treating these patients accordingly the specific infectious agent. For instance, periodontitis is a common condition in aging population, and it is associated with an increased AD risk. Therefore, this chronic inflammatory disease should be treated appropriately.

In conclusion, infections by exogeneous pathogens, insults by pollutants, and HERV activation appear to play a relevant role in the clinical history of AD. The specific treatment of these pathogens and decreasing environmental risk exposure may show beneficial effect in early intervention protocol to prevent the progression of cognitive deterioration in the elderly.

## Figures and Tables

**Table 1:** Exogenous virus (Exo virus) and endogenous retrovirus (HERV) association with human diseases of the central nervous system.

Human Disease	Exo Virus	HERVs
HIV associate neurodegeneration	HIV (clinical complications HSV-1, CMV, HHV6, EBV)	HERV-K
AD	HSV-1, HHV-6 and 7, CMV, EBV	HERV-K, HERV-H
ALS	HLTV-1 (HIV-1 weak)	HERV-K
MS	HSV-1, HHV-6 and 7	HERV-W
